# Can Blood Flow Restriction Be the Key to Reducing Quadriceps Weakness in the Early and Mid-Phases After Anterior Cruciate Ligament Reconstruction with a Hamstring Graft? A Systematic Review of Randomized Controlled Trials

**DOI:** 10.3390/diagnostics15030382

**Published:** 2025-02-06

**Authors:** Ayrton Moiroux--Sahraoui, Jean Mazeas, Marine Blossier, Maurice Douryang, Georges Kakavas, Timothy E. Hewett, Florian Forelli

**Affiliations:** 1 Orthosport Rehab Center, 95330 Domont, France; jeanmazeas@gmail.com; 2 Orthopaedic Surgery Department, Clinic of Domont, Ramsay Healthcare, @OrthoLab, 95460 Domont, France; orthosport.kine@gmail.com; 3 Department of Physiotherapy and Physical Medicine, University of Dschang, Dschang P.O. Box 96, Cameroon; douryangmaurice@gmail.com; 4 Fysiotek Spine & Sports Lab, 11635 Athens, Greece; georgios.kakavas@gmail.com; 5 Department of Physical Education and Sport Sciences, ErgoMech-Lab, University of Thessaly, 421 00 Volos, Greece; 6 Department of Orthopaedic Surgery, Marshall University, Huntington, WV 25705, USA; hewettt@marshall.edu; 7 Haute-Ecole Arc Santé, HES-SO University of Applied Sciences and Arts Western Switzerland, 2000 Neuchâtel, Switzerland; 8 Société Française des Masseurs—Kinésithérapeutes du Sport Lab, 93380 Pierrefite sur Seine, France

**Keywords:** blood flow restriction, anterior cruciate ligament reconstruction, strength, hypertrophy

## Abstract

**Background:** Injury to the anterior cruciate ligament is one of the most common knee injuries. Following anterior cruciate ligament reconstruction, strength deficits and reduced quadriceps and hamstring muscle mass are common. Traditional strengthening protocols recommend the use of heavy loads. However, following surgery, heavy-load exercises are contraindicated to protect the joint and graft. Blood flow restriction resistance training is an alternative that optimizes muscle recovery. The aim of this study was to evaluate the effects of blood flow restriction resistance training on muscle mass and strength after ACLR. **Methods:** The Pubmed, Cochrane Library, and PEDro databases were used to constitute the corpus of this systematic review. The methodological quality of the studies was assessed with the Cochrane Collaboration’s analysis grid. **Results:** Thirty-four articles were identified in the initial search, and five randomized controlled trials were included in this review. Not all studies reported significant results regarding strength and muscle mass. Two of these studies observed a significant improvement in strength associated with blood flow restriction resistance training compared with the control group. A significant increase in muscle mass was observed in one study. **Conclusions:** The blood flow restriction resistance training method shows superior efficacy to training without occlusion, yet this device has not been shown to be more effective than heavy-load resistance training in terms of muscular strength and muscle mass. Blood flow restriction resistance training shows superior efficacy in both these variables when used with low loads. However, there are still few random controlled trials on this subject, and this review presents their limitations and biases. Future research is needed on guidelines for the application of blood flow restriction resistance training in clinical populations.

## 1. Introduction

Anterior cruciate ligament (ACL) injury is one of the most common and serious knee injuries [1], with over 50,000 surgeries performed in France in 2019 [2], and around 400,000 cases annually in the U.S [3]. The risk is higher for female athletes, who are four to seven times more likely to suffer an ACL injury than male athletes at the same level [4]. Treatment depends on factors like functional instability, the type of injury, and the patient’s activity level, with surgery often recommended for young athletes [5]. Rehabilitation is key post-surgery, following a protocol that is regularly updated, most recently in 2023 [6].

A key focus is the importance of quadriceps weakness after ACLR. This weakness is often caused by neurophysiological mechanisms, particularly arthrogenic muscle inhibition, which limits the muscle’s ability to recover, even after ACLR [7,8]. This condition complicates recovery and the return to optimal functional levels [9]. To address this challenge, various rehabilitation approaches have been developed to stimulate muscle recovery and strength. These include electromyostimulation (EMS), which activates muscles through electrical impulses; vibration training, which leverages mechanical oscillations to enhance muscle contractions; aquatic therapy, which offers a low-impact environment for resistance training; and isokinetic training, which provides controlled resistance throughout a range of motion to target muscle strength. While these methods have demonstrated effectiveness in certain contexts, limitations such as access, patient adherence, or applicability during early rehabilitation phases may reduce their overall utility [6]. Blood flow restriction (BFR) emerges as a promising alternative to address this weakness. BFR devices are increasingly utilized in rehabilitation and strength training to promote muscle growth and recovery while using lower loads. Their handling requires precision to ensure safety and effectiveness. For instance, selecting the appropriate cuff size is crucial, as too narrow or wide a cuff can affect the pressure distribution. The applied pressure should be individualized based on limb circumference and arterial occlusion pressure (AOP), often determined with a Doppler ultrasound or an automated system. Devices like pneumatic cuffs or elastic bands should be positioned at the proximal part of the limb, ensuring proper placement and secure fastening without excessive constriction. Monitoring is essential during sessions to check for signs of discomfort, numbness, or vascular complications. Finally, practitioners should follow evidence-based guidelines, adjusting pressures, rest periods, and training loads according to the individual’s goals and tolerance. Proper education and adherence to protocols are vital for maximizing BFR’s benefits while minimizing risks.

**Figure 1:**
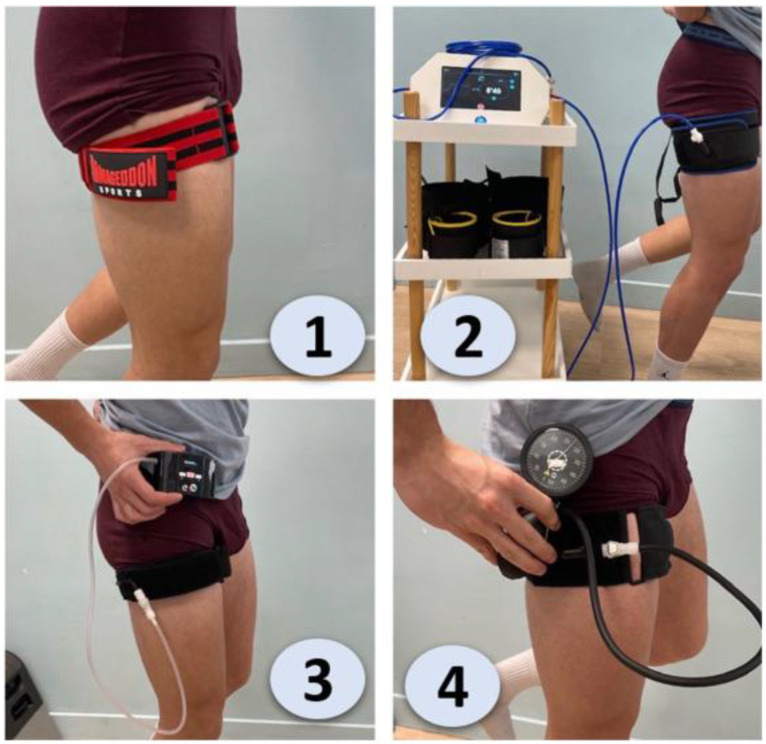
Comparison of different blood flow restriction (BFR) training devices and their application on the thigh [15]. 1. Elastic BFR (Straps or Bands), 2. Automated Pneumatic BFR, 3. Manual BFR (Pump and Gauge), 4. Personalized Pressure BFR (Integrated Gauge).

BFR, when used in combination with low-load resistance training (BFR-RT), involves application of an external compression device (such as pneumatic cuff) to a limb to restrict blood flow to the muscle, creating a hypoxic environment that induces high metabolic stress. This environment promotes muscular adaptations at low loads, effectively enabling strength and muscle growth similar to that provided by high-load training, but without the same joint stress. Meta-analyses, such as those by Hughes et al. and Patterson et al., report that BFR-RT can yield strength gains and hypertrophy comparable to that provided by high-load resistance training, with intensities as low as 20–30% of the one-repetition maximum (1RM) [10,11,12]. This approach is advantageous for ACLR patients in the early stages of recovery, as traditional high-load exercises can place undue stress on the healing graft (Figure 1).

Centner et al. reported that BFR-RT specifically enhanced quadriceps strength post-ACLR, and addressed the persistent muscle inhibition that often limits full recovery [13]. The study emphasized BFR’s role in the stimulation of protein synthesis and satellite cell activation through metabolic stress rather than mechanical load, making it a particularly valuable method for protection of the graft during early- and mid-phase rehabilitation [14]. Although further studies are needed to standardize BFR protocols and confirm long-term outcomes, current evidence supports BFR’s potential for the reduction of post-ACLR quadriceps deficits and the acceleration of functional recovery.

ACL injuries are common, and can have serious functional consequences that may restrict participation in sports. After a reconstruction, rehabilitation aims to regain muscle strength, particularly in the quadriceps and hamstring, which are most affected. Although heavy loads are effective in strengthening muscles, they are contraindicated post-operatively. BFR-RT, using low loads, is a promising alternative for promoting muscle recovery while protecting the graft. The main objective of this review was to find out whether rehabilitation using BFR-RT results in a gain in strength and muscle mass compared to traditional rehabilitation after ACLR during the early and mid-phases.

## 2. Materials and Methods

This review followed the Preferred Reporting Items for Systematic reviews and Meta-Analyses (PRISMA) 2020 statement for systematic reviews [16]. The review was not registered a priori, and no patients or members of the public were involved.

### 2.1. Eligibility Criteria

Eligibility criteria for the inclusion of articles in this literature review were defined. Inclusion criteria included a study population between 15 and 50 years old after ACLR with a hamstring graft, the intervention (rehabilitation + BFR-RT), comparison to conventional rehabilitation, assessment criteria (strength and/or muscle mass), and English language. Conversely, some criteria led to the exclusion of articles, such as systematic reviews, meta-analyses, and studies that involved participants with additional knee pathologies alongside ACLR (other concomitant ligament reconstructions).

### 2.2. Information Sources, Literature Search, and Selection of Sources of Evidence

An exploratory search was performed in the MEDLINE database using the following terms: ‘anterior cruciate ligament’ AND ‘reconstruction’ AND ‘blood flow restriction’. By this preliminary search, MeSH terms, text word terms, and relevant keywords, as well as search strategies from relevant systematic reviews, were identified, and in consultation with the authors team, the final keyword string to be used for the search was developed (Table 1).

Relevant studies were identified by the lead author by a systematic search of four online databases from January 2018 to December 2023 (MEDLINE via PubMed, Cochrane Library, and PEDro). The reference lists of the included studies and key randomized controlled trials were screened, and citation tracking was performed with Google Scholar, in order to identify any potentially relevant studies that may have been missed in the database search.

**Figure 2:**
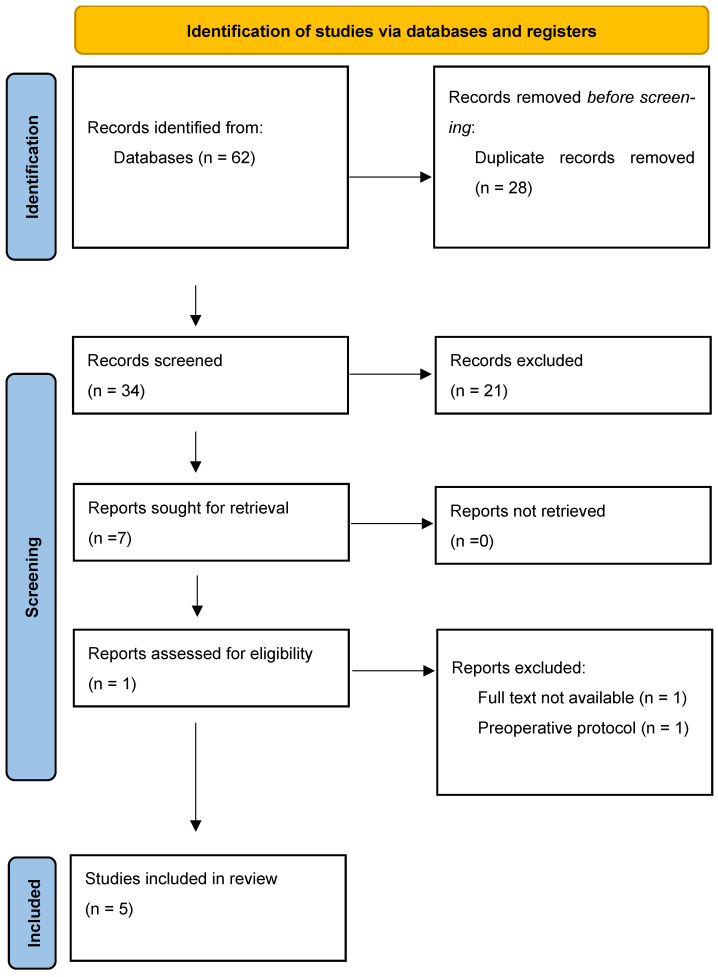
Preferred Reporting Items for Systematic Reviews and Meta-Analyses Extension for Systematic Reviews flow chart.

All articles were downloaded and transferred to the Zotero v.6.0.13 management platform. They were cross-referenced and any duplicates were deleted before the selection criteria were applied. Two reviewers (AMS and MB) independently screened all articles for eligibility by title and abstract (Figure 2). The same independent reviewers performed full-text screening to determine the final study selection. Any discrepancies were resolved during a consensus meeting first between reviewers, and if required with the participation of the senior author.

### 2.3. Data Extraction and Quality Assessment

The data extracted by AMS and MB included the names of authors, year of publication, name of article, publication journal, nationality of study, strengthening protocol used, study type, number of participants, participant characteristics, interventions used, and study results. When analyzing the articles, various data from the included studies were used. A flow chart according to the PRISMA 2020 statement was employed [16]. All articles were downloaded and transferred to the Zotero v6.0.13 management platform. They were cross-referenced and any duplicates were deleted before the selection criteria were applied. Two reviewers (AMS and MB) independently screened all the articles for eligibility by title and abstract (Figure 2). The same independent reviewers performed full-text screening to determine the final study selection. Any discrepancies were resolved during a consensus meeting, first between reviewers, and then, if required, with the participation of the senior author (Figure 2).

The validity of the identified articles was assessed with the Cochrane Risk of Bias Scale. The Cochrane Collaboration Risk of Bias Scale assesses the risk of bias in randomized clinical trials. This tool was updated in 2011. It was determined whether each article presented a risk. For each bias, it was determined whether the risk of bias was low, high, or indeterminate.

## 3. Results

After the deletion of duplicates, thirty-four articles were read. Twenty-one (62%) of them did not meet the inclusion criteria. Therefore, five were selected for inclusion in the analysis (Table 2). These five randomized controlled trials were included in this systematic review. These five randomized controlled trials directly examined the use of BFR-RT in patients who have undergone ACLR (Table 3).

Among the included studies, five compared blood flow restriction resistance training (BFR-RT) with control groups or other muscle strengthening methods (Table 1). BFR-RT protocols varied in duration, intensity, and frequency, with programs lasting from three to twelve weeks, and including between two and three sessions per week.

Most of these studies used muscle strength criteria to assess the effects of BFR-RT on the quadriceps. Erickson et al. measured quadriceps strength, reporting a difference of 39 ± 47 Nm between the BFR-RT and placebo groups [17]. Hughes et al. observed comparable increases in 10RM strength between the BFR-RT and HL-RT groups (104 ± 18% vs. 106 ± 21%, *p* = 0.22), although a significant increase in isokinetic strength was noted in the BFR-RT group at speeds of 150°/s and 300°/s (*p* < 0.001) [18].

Regarding muscle mass criteria, three studies measured the quadriceps cross-sectional area or muscle thickness. Erickson et al. reported a 27 ± 32 cm^2^ increase in muscle area in the BFR-RT group compared to placebo after four months of training [17]. However, Hughes et al. found no significant difference between BFR-RT and HL-RT in terms of quadriceps muscle thickness increase (5.8 ± 0.2% vs. 6.7 ± 0.3%, *p* = 0.33) [18].

Three studies (60%) used both strength and endurance criteria to assess the effects of BFR-RT, including evaluations of isokinetic strength, 1RM, and muscle contraction. Kacin et al. reported a significant increase in knee extensor strength in the LL-BFR-RT group at 60°/s (14 ± 11% vs. 0 ± 6%, *p* < 0.05) and 120°/s (8 ± 5% vs. −2 ± 5%, *p* < 0.01), compared to the control group [20].

### Data Quality

The risk of bias varied across studies. The study by Erickson et al. showed a low risk of bias in random sequence generation, allocation concealment, detection, migration, and notification. However, this study showed performance bias, and other sources of bias were also present [17]. The study by Hughes et al. had a low risk of bias in random sequence generation, allocation concealment, and notification. However, this study had performance and migration biases, and the detection bias remains undetermined [18]. The study by Curran et al. showed a low risk of bias in random sequence generation, allocation concealment, and notification, but had detection and performance biases [19]. Migration bias was undetermined in this study. The study by Kacin et al. had a low risk of bias in random sequence generation and allocation concealment, but presented performance and detection biases [20]. Migration and notification biases were undetermined. Finally, the study by De Melo et al. had a low risk of bias in random sequence generation and allocation concealment, but performance and detection biases were observed [21]. Migration and notification biases were undetermined (Table 4).

## 4. Discussion

The present systematic review aimed to evaluate the efficacy of BFR-RT in improving quadriceps strength and muscle mass in patients after ACLR. The findings show that BFR-RT is a promising adjunct to traditional low-load training protocols, and offers a significant alternative for early- and mid-phase rehabilitation by enabling muscle hypertrophy and strength gains with reduced joint strain. This review reveals a nuanced view of the efficacy of BFR-RT in enhancing quadriceps strength and muscle mass after ACLR. Several studies within this review underscore the potential of BFR-RT for fostering strength gains that are comparable to, and in some cases greater than, those achieved with traditional HL-RT. However, the results show variability across studies, which indicates that BFR-RT’s effectiveness is influenced by specific protocol variables, such as load intensity, cuff pressure, and timing of the intervention post-surgery.

The current review highlights multiple studies in which BFR-RT led to significant increases in quadriceps strength. For instance, Hughes et al. demonstrated that BFR-RT using loads of 30% of the 1RM with occlusion at 80% of the LOP achieved strength gains comparable to those provided by HL-RT performed at 70% of the 1RM. This finding shows that BFR-RT can match the effectiveness of higher-load training, a notable advantage in the early stages post-ACLR, when high mechanical loads are generally contraindicated [18]. Furthermore, Kacin et al. reported that BFR-RT resulted in greater gains in isokinetic strength at both 60°/s and 120°/s compared to a control group without BFR [20]. This improvement in both maximum torque and total work performed emphasizes BFR-RT’s efficacy in building strength across different speeds of muscle contraction, which is particularly beneficial for functional recovery in ACLR patients.

There are studies in this current review, such as that by Curran et al., that showed no additional benefit of BFR-RT when combined with high-intensity exercise (e.g., 70% of 1RM) [19]. This indicates that BFR-RT may be most effective when applied in a low-load context rather than in conjunction with heavy loads, potentially due to the increased metabolic stress and hypoxic conditions specific to low-load BFR-RT protocols, which stimulate muscle adaptations without the need for high mechanical loads. The present review also shows that BFR-RT can be effective for the promotion of muscle hypertrophy post-ACLR, although the results here are mixed. Erickson et al. observed a notable increase in quadriceps cross-sectional area in the BFR-RT group, with a 27 ± 32 cm^2^ difference compared to a placebo group. This finding aligns with the hypothesis that BFR-RT promotes muscle hypertrophy through mechanisms associated with metabolic stress, such as increased muscle protein synthesis and satellite cell proliferation, even under low-load conditions [17]. Similarly, Kacin et al. found that low-load BFR-RT significantly increased quadriceps muscle mass compared to a control group [20]. This study’s protocol involved four sets of knee flexion and extension at 40% of the 1RM with occlusion pressure set at 150 mmHg, underscoring the potential for specific BFR-RT configurations to achieve hypertrophy. The significant gains in quadriceps cross-sectional area achieved in this study show that BFR-RT is particularly effective when combined with moderate occlusion pressures and moderate training loads, potentially optimizing the balance between metabolic stress and muscle activation for hypertrophy.

Other studies did not find a statistically significant difference in muscle mass between BFR-RT and non-BFR-RT groups. For instance, Curran et al. measured rectus femoris mass, but found no meaningful increase in the BFR-RT group compared to controls, indicating that the effects of BFR-RT on hypertrophy may vary based on muscle group, training duration, and protocol parameters [19]. Hughes et al. also reported similar muscle thickness increases in both BFR-RT and HL-RT groups, which indicates that BFR-RT may not universally exceed the hypertrophic effects of traditional high-load training, but offers a viable alternative in situations where high loads are unsuitable [18].

In summary, the results of this current review support BFR-RT as a beneficial method for achieving muscle strength and mass gains in early- and mid-phase rehabilitation after ACLR. BFR-RT has demonstrated comparable efficacy to HL-RT in strength development, particularly in protocols involving low to moderate loads. This is significant, given the need to limit mechanical load on the knee joint in the early stages of recovery. The variability in hypertrophy outcomes across studies shows that BFR-RT’s effect on muscle mass may be influenced by factors such as cuff pressure, load intensity, and intervention timing. Further research with standardized protocols is warranted to optimize BFR-RT application for consistent hypertrophic outcomes.

### 4.1. Limitations

A potential limitation of this systematic review is that it was not registered on PROSPERO or a similar registry, which could enhance transparency and adherence to predefined protocols; however, this will be addressed in future research endeavors. The heterogeneity in study protocols presents a limitation in drawing generalized conclusions regarding BFR-RT’s efficacy. Variability in factors such as cuff pressure, load intensity (from 30% to 70% of 1RM), and training duration may influence outcomes and limit comparability across studies. For example, Erickson et al. included a preoperative BFR-RT protocol, whereas other studies commenced several weeks post-surgery, likely affecting muscle recovery pathways and adaptation rates [17]. Another limitation is the small sample sizes across the included studies, which limits statistical power and may bias the results. In addition, the lack of blinding in most trials presents a risk of performance and detection bias. Participants and assessors in studies such as those by Hughes et al. were not blinded, potentially influencing subjective assessments of strength and hypertrophy [18]. One of our limitations is the small number of studies included, because we selected studies from the last 5 years.

### 4.2. Clinical Implications and Perspectives

**Figure 3:**
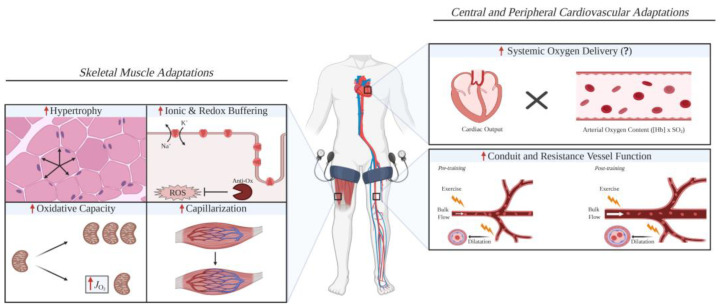
A schematic diagram illustrating the possible skeletal muscle and cardiovascular adaptations that are improved with blood flow restriction training compared with work-matched training [22].

Despite these limitations, the present review supports the clinical potential of BFR-RT as a low-load alternative in the early rehabilitation of ACLR patients, where traditional heavy-load training is contraindicated. Its application could potentially improve patient adherence by reducing the pain associated with high-load exercises, facilitating muscle recovery (Figure 3). However, to translate BFR-RT into standard clinical practice, further research is needed to establish standardized protocols that define the optimal cuff pressure, load intensity, and duration of application for ACLR patients. Future studies with larger, more homogenous sample populations and robust blinding methodologies are necessary to confirm BFR-RT’s long-term efficacy and safety. Investigation of the biochemical and physiological mechanisms underlying BFR-induced hypertrophy, such as BFR’s effects on protein synthesis and satellite cell activity, would also provide insights into its optimization of its use in post-operative rehabilitation.

## 5. Conclusions

BFR-RT shows promise as an effective method for the improvement of quadriceps strength and muscle mass in the early- and mid-rehabilitation phases after ACLR. BFR-RT allows strength gains that are comparable to those provided by traditional high-load resistance training, but with lower intensities, reducing strain on the healing knee joint. This makes BFR-RT particularly valuable for patients who cannot yet safely undertake heavy-load exercises post-surgery. This current review indicates that BFR-RT can effectively foster muscle hypertrophy and strength; however, results vary across studies. This variability may stem from differences in load intensity, cuff pressure, and protocol timing, which highlights a need for standardized guidelines to optimize BFR-RT application. Limitations in current research, such as small sample sizes and a lack of standardized methodologies, underscore the importance of future studies to confirm these findings and establish best practices. In conclusion, BFR-RT is a promising addition to ACLR rehabilitation, which offers a safe and effective way to mitigate muscle weakness. Further research is essential to validate its benefits and refine protocols, in order to ensure its integration into clinical practice for optimal patient outcomes.

## Figures and Tables

**Table 1:** Search terms.

Construct	Keywords
Population	Anterior cruciate ligament reconstruction, Anterior cruciate ligament reconstruction graft, Anterior cruciate ligament reconstruction surgery
Concept	Blood flow restriction, Blood flow restriction therapy, Blood Flow Restriction Training
Context	Muscular strengthening, Resistance training, Strength training, Strengthening program

**Table 2:** General characteristics of included studies.

References	Study Title	Aim of the Study	Country of Study	N (Participants)
Erickson et al. [17] (2019)	Effect of Blood Flow Restriction Training on Quadriceps Muscle Strength, Morphology, Physiology and Knee Biomechanics Before and After Anterior Cruciate Ligament Reconstruction	Evaluate the effect of BFR-RT on quadriceps strength and knee biomechanics, and identify the potential mechanism(s) of action of BFR-RT at the cellular and morphological levels of the quadriceps.	USA (University of Kentucky)	A total of 60 patients (male or female, 15–40 years) with an isolated ACL tear or with a meniscus tear, without a specified graft type. They were randomly assigned to the following groups: - Physiotherapy + BFR (n = 30) (BFR-RT group) - Physiotherapy + BFR placebo (n = 30) (standard of care group)
Hughes et al. [18] (2019)	Comparing the Effectiveness of Blood Flow Restriction and Traditional Heavy Load Resistance Training in the Post-Surgery Rehabilitation of Anterior Cruciate Ligament Reconstruction	Compare the efficacy of BFR-RT and traditional heavy-load training (HL-RT) in improving skeletal muscle hypertrophy and strength, physical function, pain, and effusion in patients undergoing anterior cruciate ligament reconstruction.	UK (National Health Service)	A total of 28 patients (11 women and 17 men, age: 29 ± 7 years) underwent unilateral autograft surgery with a graft from the hamstrings. They were randomly assigned to the following groups: - HL-RT (n = 14) - BFR-RT (n = 14)
Curran et al. [19] (2019)	Blood Flow Restriction Training Applied with High-Intensity Exercise does not improve Quadriceps Muscle Function After Anterior Cruciate Ligament Reconstruction	Examine the efficacy of BFR-RT with high-intensity exercise on the recovery of quadriceps muscle function in patients after anterior cruciate ligament reconstruction.	USA (University of Michigan)	A total of 34 patients (19 women, 15 men, age: 16.5 ± 2.7 years) who had undergone ACLR were randomly assigned to one of four groups: - Concentric (n = 8) - Eccentric (n = 8) - Concentric + BFR-RT (n = 9) - Eccentric + BFR-RT (n = 9)
Kacin et al. [20] (2021)	Functional and molecular adaptations of quadriceps and hamstring muscles to blood flow restricted training in patients with ACL rupture	Determine whether LL-BFR-RT can increase motor function and the size of quadriceps and hamstring muscles in patients after ACL reconstruction.	Slovenia (Ljubljana)	A total of 18 participants (9 women and 9 men, age: 37.5 ± 9 years) underwent ACLR. A total of 12 people were divided into three groups: - LL-BFR-RT(n = 6) - Control group (n = 6) - No-intervention control group (n = 6), used for muscle biopsy analysis.
De Melo et al. [21] (2022)	Comparison of Quadriceps and Hamstring Muscle Strength After Exercises with and without Blood Flow Restriction Following Anterior Cruciate Ligament Surgery	Compare quadriceps and hamstring muscle strength gain in patients after ACL reconstruction surgery using exercises with and without BFR-RT.	Brazil (University of São Paulo)	A total of 28 participants (male and female, age: 18–59) underwent ACLR using a hamstring autograft.The were randomly assigned to the following groups: - BFR-RT group (n = 14) - Control group (n = 14)

Note: ACL, anterior cruciate ligament; BFR, blood flow restriction.

**Table 3:** Overview of BFR-RT intervention protocols and main findings in ACLR studies.

References	Duration	nterventions	Main Findings
Erickson et al. [17]	- Pre-surgical: 3X/week for 4 weeks - BFR-RT post-surgery: Start 1 month before surgery and resume at 2 weeks post-op. 3X/week for 4–5 months. Sessions last 20 min. - Standard post-surgery: 3X/week for 6–7 months, divided into three stages (3 days–2/6 weeks, 4–6 weeks, 3–5 months). Home-based program provided.	BFR-RT: Stopped 4 months post-surgery for isolated ACL, or 5 months if meniscus repair is included. Pressure defined by manufacturer. Placebo: Minimal pressure (<20 mm Hg). All participants focused on quadriceps strengthening.	- Quadriceps strength: Difference of 39 ± 47 Nm between groups. - Muscle cross-sectional area: Difference of 27 ± 32 cm^2^ between groups.
Hughes et al. [18]	8 weeks 2X/week (16 sessions), beginning on day 14 post-op	BFR-RT group: - Warm-up: 5 min cycling, 10 reps unilateral press with light load. - Unilateral press at 30% of 1RM with 80% of LOP, four sets (30, 15, 15, 15 reps) with 30 s rest and continuous BFR. HL-RT group: Same warm-up and unilateral press at 70% of 1RM, three sets of 10 reps with 30 s rest. Both groups followed a standard rehab program 3X/week at home.	- 10RM strength: Significant increase in both groups (BFR-RT: 104 ± 18%; HL-RT: 106 ± 21%, *p* < 0.01), with no difference between groups (*p* = 0.22). - Kinetic force: Torque decrease in both; BFR-RT group—significant increase at 150°/s and 300°/s. - Muscle mass: Significant increase (BFR-RT: 5.8 ± 0.2%, HL-RT: 6.7 ± 0.3%, *p* < 0.01), with no difference between groups (*p* = 0.33).
Curran et al. [19]	8 weeks 2X/week (16 sessions), starting 10 weeks post-op	All participants followed standard rehabilitation. 1RM assessed on first day, then weekly. Experimental (BFR-RT): five sets of 10 reps at 70% of 1RM unilateral leg press with 2 min rest between sets; cuff deflated during rest and reinflated for sets. Pressure: 80% of LOP (110–186 mmHg). Control: Same exercises and intensity without occlusion.	- Kinetic force: BFR-RT = −12.4 ± 19.2 Nm vs. Control = −15.0 ± 19.2 Nm, *p* = 0.49. - Isometric strength: BFR-RT = −16.3 ± 31.1 Nm vs. Control = −11.8 ± 38.3 Nm, *p* = 0.88. - 1RM: BFR-RT = 2.44 ± 1.21 kg vs. Control = 2.10 ± 0.95 kg, *p* = 0.24. - No statistical difference (*p* < 0.05) for isokinetic, isometric extension, and rectus femoris muscle mass.
Kacin et al. [20]	3 weeks 3X/week for nine sessions	BFR-RT group: four sets of knee extension and flexion at 40RM until failure with operated leg only, workload constant. Cuff pressure: 150 mmHg. Isotonic knee extension with 45 s rest between first and third sets without reperfusion; 90 s reperfusion after second set. Same protocol for knee flexion. Placebo: Same protocol with cuff inflated to 20 mmHg.	- Knee extensors: Higher max torque at 60°/s (14 ± 13% vs. −1 ± 7%), total work at 60°/s (14 ± 11% vs. 0 ± 6%), max torque at 120°/s (10 ± 9% vs. −4 ± 7%), total work at 120°/s (8 ± 5% vs. −2 ± 5%) in BFR-RT group. - Knee flexors: Max torque similar in both groups. - Muscle cross-sectional area: Significantly higher for quadriceps in BFR-RT group (5.0 ± 3.4%) than placebo (1.1 ± 2.1%); hamstring similar between groups.
De Melo et al. [21]	12 weeks 2X/week	BFR-RT group: four sets (1×30, 3X15), with 30 s rest between sets, at 30% MR. Pressure: 80% of LOP. Control group: three sets of 10 reps at 70% of 1RM. Pressure maintained during all reps. 1RM tests conducted on leg press and flexion machine for BFR training on injured then uninjured leg.	- Knee extensor strength: Increase throughout cycle for both; greater gain in BFR-RT from second post-op period, with significant difference after 12 weeks. No difference in uninjured legs. - Knee flexor strength: Increase throughout cycle for both, greater gain in BFR-RT from third post-op period. No difference in uninjured legs.

**Table 4:** Risk of Cochrane bias in various studies.

	Erickson et al. [17]	Hughes et al. [18]	Curran et al. [19]	Kacin et al. [20]	De Melo et al. [21]
Randomization sequence generation	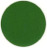	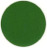	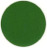	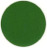	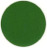
Allocation concealment	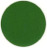	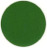	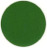	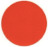	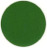
Performance biases	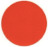	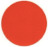	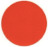	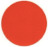	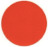
Detection biases	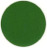	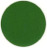	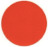	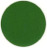	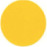
Migration biases	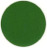	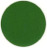	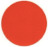	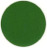	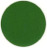
Notification biases	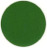	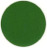	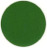	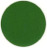	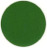
Other sources of bias	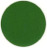	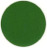	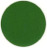	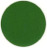	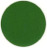

Low risk of bias: 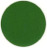. High risk of bias: 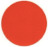. Risk of bias undetermined: 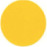.
